# Leafy green crops to improve diets on Pacific islands 

**DOI:** 10.2471/BLT.18.020918

**Published:** 2018-09-01

## Abstract

Cultivating leafy greens locally is one of the many approaches being taken to tackling noncommunicable diseases in the Pacific Islands. Georgina Kenyon reports.

People in Kiribati speak nostalgically about traditional Sunday lunch at their grandparents’ with lots of fresh fish wrapped in taro leaves and cooked with fresh, tasty vegetables.

Now, fruit and vegetables are more likely to be flown in from Australia than grown locally.

Eating has changed on these islands, along with the climate.

On Kiribati and in other Pacific Island states, there is a high burden of noncommunicable diseases (NCDs) such as heart disease, stroke and diabetes. An estimated 70% of deaths in Pa­cific Island countries are due to these diseases.

The proportion of the population in Pacific countries affected by NCDs is expected to continue to rise due to population growth and ageing, unless countries tackle risk factors such as tobacco use, inadequate physical activity, and unhealthy diet.

Healthier diets can help reduce the main risk factors for NCDs: high blood pressure, obesity, high blood sugar and high cholesterol. 

In 2015, governments committed to reducing premature mortality from NCDs by one third, under SDG3 on health. They recognized that this could not be achieved by the health sector alone and that other sectors – such as agriculture, trade and finance – must be involved. 

Efforts to improve diets in Kiribati and the Marshall Islands, Tuvalu and Tokelau, are a case in point. 

It is hard to grow crops on coral atolls, but volcanic islands often have rich soil, where fruit and vegetables grow more easily. 

“Growing a wide range of crops on coraline soils has always been challeng­ing, but climate change is compounding the issue due to unpredictable rainfall and more frequent drought,” says Graham Lyons, a researcher in plant and food science at the University of Adelaide in Australia. 

Kiribati, a chain of 33 islands, has a population of about 114 000. “The atoll nature of Kiribati makes it very difficult to grow most root crops, fruits and vegetables, so only a limited variety of foods can be grown,” says Dr Ezekiel Nukuro, World Health Organi­zation (WHO) Country Liaison Officer in Kiribati. 

 “Most people can’t afford imported fruit and vegetables, and climate change impacts, such as drought, sea level rise resulting in coastal erosion and increased salinity of soil, makes the situation even more difficult.

“Climate change impacts, such as drought, sea level rise resulting in coastal erosion and increase salinity of soil, make the situation even more difficult.”

Ezekiel Nukuro

“Ensuring food security and promoting the growth of local foods is among the government’s development priorities here,” Nukuro says.

That’s why Lyons has been advising Kiribati’s government on crops for the last two years and promoting plants such as chaya, amaranth, kangkong, beach cowpea and purslane.

“Leafy plants that few people eat and some regard as weeds, hold immense potential for improving the diets of local people,” he says.

Lyons is involved in a project funded by the Australian Centre for International Agricultural Research to improve soil quality and nutrition security on the outer islands and atolls of Kiribati and Tuvalu.

“The benefits of particular leafy greens in the Pacific Islands have long been known,” says Dr Si Thu Win Tin, Team Leader for NCDs in the regional office of the Pacific Community in Fiji. “The Pacific Community produced a book, *The leaves we eat,* that provides information on nutrient contents and tips on how to grow, harvest and prepare such plants.”

The Pacific Community also produced a video series entitled *Edible leaves in the Pacific* and is providing technical support to small island developing states, including to promote cultivation of such crops in a sustainable way.

Nutritious green leafy plants such as chaya were introduced to the islands from Mexico and other countries in the 1980s. Now they are flourishing on many Pacific islands.

Currently, Kiribati is importing staple foods such as rice and flour, also fruits and vegetables to meet its population needs and demands.

The idea is that by replacing imported processed foods that are often high in fats, salt and sugars with local leafy or root vegetable produce and promoting physical activity, people will become healthier and NCDs can be reduced.

“The United States Navy did an epidemiological survey throughout Micronesia at the end of the Second World War and found a very low incidence of diabetes.

“About 15 years later, after people had started eating unhealthy imported foods, NCDs were well established,” Lyons says.

Improving diets is one of the many approaches for preventing NCDs outlined in the *NCD roadmap report*, developed by the World Bank in 2014.

Core strategies include stepping up tobacco control, reducing consumption of food and drink products directly linked to obesity, heart disease and diabetes and investing more in the approaches recommended in WHO’s *Package of essential noncommunicable disease interventions for primary health care in low-resource settings*.

“Good primary health care is essential for identifying risk factors and NCDs in their early stages and to provide appropriate interventions to prevent complications,” says Dr Cherian Varghese, Coordinator for the Management of NCDs at WHO headquarters in Geneva. 

In Kiribati and Tuvalu, Lyons and his colleagues are working with the agriculture and health ministries as well as community groups and Island Councils to grow leafy vegetables, such as taro, sweet potato, papaya, watermelon and beans, and to build traditional food gardens, known as giant swamp taro pits. These pits are dug one to two metres deep to reach the freshwater on the islands.

Meanwhile, local cooks are helping people to adapt traditional recipes to suit this new, green diet.

Kiribati and other Pacific islands have come a long way in the past 10 years in terms of awareness-raising to encourage healthy eating in general.

Today many Pacific islanders are more aware of the need to keep salt intake low and to read the nutritional label on packaged food as a guide before buying a product.

Taxes on foods high in fat and salt are also being used by Pacific governments.

“Following the success of efforts to reduce tobacco and alcohol consumption in the Pacific Island states, these countries have increased taxes on selected products such as high-fat processed meats, instant noodles, sugar-sweetened beverages and other high-sugar foods,” says Wendy Snowdon, WHO’s Advisor on NCDs in the Pacific Islands.

At the same time, some countries have reduced taxes on products, such as fruits and vegetables, to encourage their consumption.

“The impact of food taxes on consumer behaviour is still being assessed, but the indications are positive for many of the taxes, and of course taxes are intended to complement other measures such as education, policy change and community mobilization,” Snowdon says.

“Taxes are unlikely to have an impact in isolation, but should be introduced as part of a suite of interventions to improve diets,” she adds.

Governments can also take action to reduce NCDs by tightening food-related legislation, by supplying educational and advocacy programmes and by improving diets in schools.

But efforts to take such a multisectoral approach – also envisaged in the 2030 agenda for sustainable development – can be challenging, given the conflicting interests of diverse sectors.

For example, in 2007, Samoa imposed a ban on the import of turkey tails – a cheap fatty meat product – to help combat its obesity epidemic.

In 2011, the island state lifted the import ban as a condition for joining the World Trade Organization, increasing global concerns that trade agreements were stifling policy innovations. 

Samoa was, however, allowed to introduce a sales ban for two years, along with a 300% sales tax on turkey tails to address health concerns.

Snowdon also stresses the importance of encouraging healthy behaviours from a young age, and ensuring that schools are settings where youth can learn and practice healthy lifestyles.

While NCDs remain a major challenge in the Pacific, there are signs that diets might be changing.

In Papua New Guinea and the Solomon Islands, for example, people now eat plenty of aibika, a green leafy plant, sometimes compared to spinach.

“People in the Pacific Islands changed their eating habits quickly when exposed to the worst of western and eastern processed foods,” Lyons says.

“Now they need to change again, go back to what they did before and add new crops to improve on that.”

**Figure:**
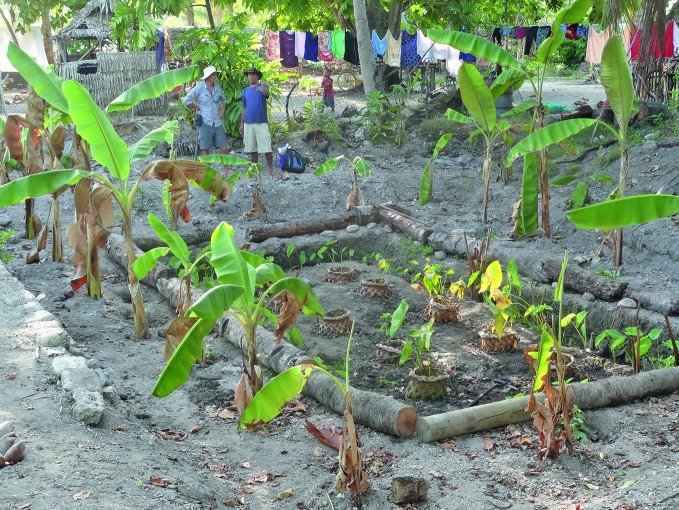
Giant swamp taro pit on Abemama atoll, Kiribati.

Graham Lyons

**Figure:**
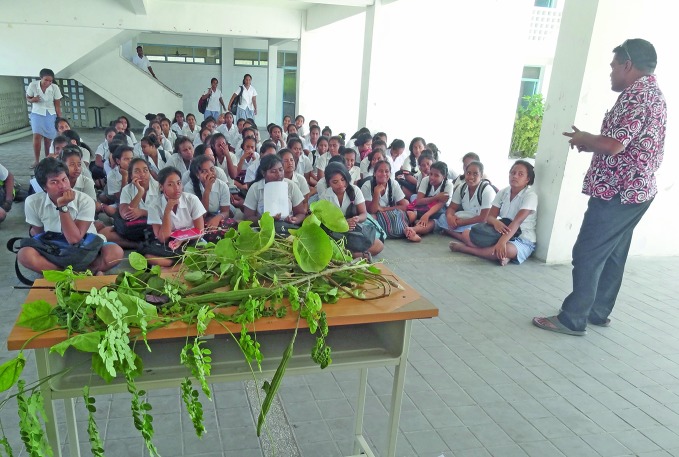
Students at King George V School in Tarawa atoll, Kiribati learn about the importance of including local nutritious leafy vegetables in their diets.

Graham Lyons

